# Unsupervised SAR Imagery Feature Learning with Median Filter-Based Loss Value

**DOI:** 10.3390/s22176519

**Published:** 2022-08-29

**Authors:** Krzysztof Gromada

**Affiliations:** Institute of Automatic Control and Robotics, Warsaw University of Technology, A. Boboli 8 St., 02-525 Warsaw, Poland; krzysztof.gromada.dokt@pw.edu.pl

**Keywords:** Synthetic Aperture Radar (SAR), Convolutional Neural Networks (CNN), Deep Neural Networks (DNN), autoencoders (AE), transfer learning, Automatic Target Recognition (ATR)

## Abstract

The scarcity of open SAR (Synthetic Aperture Radars) imagery databases (especially the labeled ones) and sparsity of pre-trained neural networks lead to the need for heavy data generation, augmentation, or transfer learning usage. This paper described the characteristics of SAR imagery, the limitations related to it, and a small set of available databases. Comprehensive data augmentation methods for training Neural Networks were presented, and a novel filter-based method was proposed. The new method limits the effect of the speckle noise, which is very high-level in SAR imagery. The improvement in the dataset could be clearly registered in the loss value functions. The main advantage comes from more developed feature detectors for filter-based training, which is shown in the layer-wise feature analysis. The author attached the trained neural networks for open use. This provides quicker CNN-based solutions implementation.

## 1. Introduction

The rapid development of Artificial Intelligence (AI) and Convolutional Neural Networks (DNN) [1] in particular led to the creation of multiple immense databases for supervised learning. Most popular datasets, such as ImageNet [2], MS COCO [3], CIFAR [4], focus on optical images in color or grey-scale. Other popular types of images with numerous datasets are medical images (e.g., COVID-19 V7 database [5]) or Infrared Imagery (e.g., FLIR [6]).

With open datasets also comes a variety of pre-trained neural networks (YOLO series [7], Resnet50 [8,9], R-CNN [10]). Big datasets can also be used to extract sets of features that enable further implementation as part of DNN or statistical algorithms for image analysis.

Recently Synthetic Aperture Radar (SAR) systems have gained increasing popularity. These devices enable the monitoring of large areas of the earth’s surface from satellites [11,12,13], airplanes or UAVs [14]. One of the main advantage of SAR technology is its high resistance to environmental conditions. However, due to entirely different work principles than optical devices, these images carry immense amounts of hard-to-extract information.

Proper processing of the images created by SAR can generate unique information on the terrain. Interferometry [12] facilitated the monitoring of ground activity (e.g., volcano area seismic changes [15]), while Coherent Change Detection algorithm helps in the detection of sub-wavelength object deformations [16] (e.g., car wheel traces), and histogram-based algorithms can provide a tool for image semantic segmentation [17]. Among AI solutions, a recent article investigated Deep Neural Networks for crop classification [11,18,19].

Despite the common usage of SARs, due to their governmental or military usage rather than civilian or commercial, few datasets exist for AI training (e.g., SpaceNet6 [20], SSDD [21], MSTAR [22]). An additional reason for their sparsity might be related to the complexity of the SAR classification/segmentation task for humans, which makes it almost impossible to create a community-driven labeled dataset. Furthermore, no open-source image features are available for SAR imagery. This means that every classification/detection/segmentation/image stitching task requires the creation of a new neural network or finding a new set of features (Bag Of Words).

This article presents various unsupervised and self-supervised learning techniques for DNN pretraining in feature detection for transfer learning or further analytical processing. The article’s contribution can be divided into the following aspects:

- A novel filter-based labeling algorithm for loss calculation for CNN Pretraining;
- An open-access Bag Of Words for SAR imagery and a set of complete Neural Networks.

The first goal was to provide a novel approach for better autoencoder pretraining, which can be later used for transfer learning. As a result, neural networks trained using filtered images for loss function calculation perform better. Lower loss values were obtained for validation with and without filtering the validation label images.

The second goal was to provide faster development of Computer Vision algorithms for researchers and engineers. The performed experiments consist of training a set of Convolutional Neural Networks with different depths for different applications.

The article starts with the definition of a set of lossless data augmentation methods for dataset enhancement. Then, the description of supervised, self-supervised, and unsupervised learning methods concerning the SAR imagery is presented. Section 2.6 presents the novel filtering algorithm for autoencoders training. Then, dataset and loss values are described, and the results are presented. The results contain the loss metrics values achieved by the presented neural networks, features extracted, convolutional layers kernels, and the juxtaposition of input–output imagery for four networks. The conclusion summarizes the results, defines the advantages gained through the described methods, and proposes future development possibilities.

## 2. Neural Networks Training for SAR Imagery

Due to the minimal amount of open data labeled SAR datasets, it is crucial to determine possible ways to increase the amount of training data with data augmentation methods.

### 2.1. Data Augmentation Methods

Before applying data augmentation, it is essential to study the characteristics of the image and define a set of acceptable data augmentations operations that will not affect the image’s logic. The SAR work principle is described in [23], with interferometry in [12] or in short in [17].

One of the most apparent characteristics of the SAR scans is their isotropy—each scan can be viewed from any angle, as it is a projection of the electromagnetic wave measurements on the earth’s surface. The position of the scan and its shape do not depend on the position of the sensor as is the case for optic cameras. Due to the application of Synthetic Aperture Radars in GEOINT (Geospatial Intelligence), they are usually attached with georeferencing and metadata. They contain the parameters and external conditions from the scanning period (mainly the radar’s positions, speed, and wavelengths used).

The scan does not clearly define the position of the airplane or satellite carrying the SAR. It can be deduced by the existence of the shadow when a high man-made object or natural objects with significant height changes are present—object such as a single tree in the field or canyon-like rock formations (more in Section 2.3).

**Figure 1:**
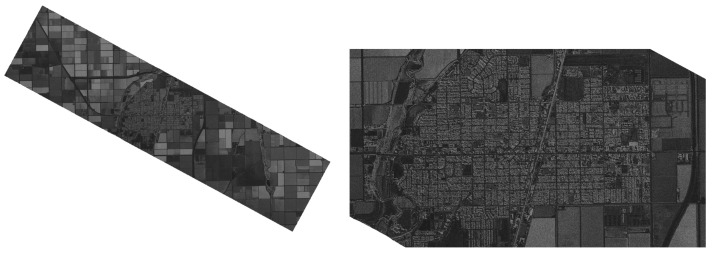
Example SAR image from 115.5° W 32.9° N WGS84 (Brawley US) from Capella Open Data, with zoom on the city in the middle.

**Figure 2:**
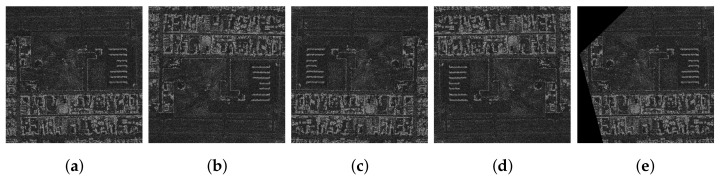
Operations of mirroring and trimming of a SAR scan. Image (**a**) presents the original image for mirroring, (**b**–**d**) are mirrored, (**e**) presents simple image trimming for data augmentation.

Thus images can be mirrored in both axes without affecting the image logic (which is not valid for most optical images) or being rotated. Additionally, since most of the SAR images contain a black frame around the image, sometimes with a smooth transition, but most often with rapid cut-off rims (Figure 1), images can be trimmed off the sides for the additional expansion of the image database. Examples of these operations are shown in Figure 2.

Since SAR imagery is a projection of the reflected radar signal on the ground, it can be rotated by any angle and still maintain its logical integrity (there is no way to determine the “bottom”, which is easy for most photographs). It is similar to nadir aerial photography, or orthophoto [24]. This means that rotation prediction cannot be used for pretext tasks for Self Supervised Learning. However, SAR imagery has a low Signal To Noise Ratio compared to optical photographs, which leads to low resolution (for satellite imagery, usually around 3–5 m to the highest of 25 cm/pix [25]). Rotation by an angle other than a multiple of 90° can lead to unwanted image blurring/aliasing.

**Figure 3:**
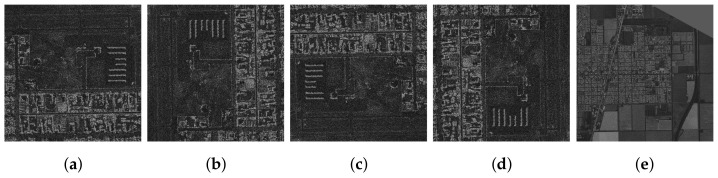
Example of rotation variants (**a**) 0°, (**b**) 90°, (**c**) 180°, (**d**) 270°and (**e**) zooming out ×5.

A similar effect occurs for zooming in or out. Only zoom-out should be performed with the natural scaling coefficient to maintain the information integrity. Examples of rotation and scaling are shown in Figure 3. The down-scaling is performed using bilinear interpolation.

For further data augmentation, two basic methods can be used—artificial noise application over the image or amplitude/histogram operations (normalization, etc.). However, these two methods can affect the image’s logic.

### 2.2. Supervised Training

For feature extraction, there are a few state-of-the-art DNN models. Among them are Siamese Networks (Triplet Loss), or Metric Learning (similarity search). They facilitate the generation of high-quality features for transfer learning. The drawback of these networks is the need for proper image labeling and preprocessing by human operators.

Due to the particular characteristics of the SAR scans mentioned above, it is hard to design a pretext task (goal of the AI to learn that does not require manual labeling).

### 2.3. Self-Supervised Learning Techniques

**Figure 4:**
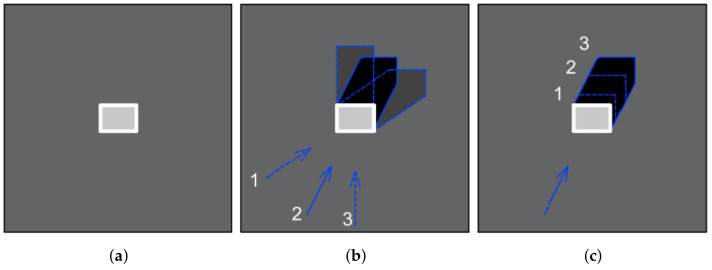
Image of a building (**a**) from close to nadir angle, (**b**) from constant Look-down/elevation angle and three azimuth angles, (**c**) from constant azimuth with different look-down angles (1 is the highest, 3 is the lowest).

For some applications, when scans are taken from off-nadir angles (look-down-angle ≠90°), a good pretext task could be for a neural network to predict the scan angle or the azimuth angle of the satellite position during the scanning process. The information about it is contained in the metadata of the scan files. The main problem with such an approach is the fact that such a pretext task could lead to focusing only on buildings and other high, man-made (usual steel) objects and their shadows. The task itself requires imagery containing high objects and it is difficult even for human operators to deduce from the imagery. An example is shown in Figure 4.

It is worth mentioning that the illustrations in Figure 4 skip the phenomenon related to the creation of the shadow area on the side of the radar. In addition, the image presents a single, high building, which is a perfect object for angle determination.

An alternative approach could be the resolution prediction task. The SAR imagery rescaling heavily alters the contained information. Due to this effect, scaling can only be performed by a total number. Thus, the image can only be presented in basic image resolutions and multiples. Due to the limited number of potential output values and the complexity of the task, it is not an effective pretext task.

Therefore, the first suggestion, i.e., azimuth angle determination, is better suited as the angle can be a floating point value <0°, 360°). The neural network would be able to focus on shadows and high objects for such a task. This can be advantageous, as shown in [26].

For such pretext tasks, a Self-supervised learning (SSL) algorithm such as SESEMI [27] can be used. In this research, no SSL technique was used for the reasons mentioned above.

It is worth mentioning the novel approach to “Unsupervised self-learning” presented by Ben Abbes A. and Jarray N. [28]. It is able to achieve very good results but requires the training of a second model and works on correlated scan images, which makes the approach difficult to perform in a real environment. Especially when considering airplane carriers rather than satellites.

### 2.4. Unsupervised Learning Techniques

Among the most popular unsupervised learning techniques for images, there are exist autoencoders [29], sparse coding networks [30], and Generative Adversarial Networks (GANs) [31]. Additionally, algorithms such as the Boltzmann Machine or Deep Belief Networks can be adapted for image processing.

### 2.5. Autoencoders

SAR imagery usually has high noise levels. Thus, it seems very practical to train a denoising autoencoder for scan filtering [32].

For the sole purpose of feature extraction, it is essential to maintain knowledge of the pixel brightness outliers as they may contain important information. For example, a single bright spot can represent a car with a 3 m × 3 m pixel size. Thus, a neural network must consider all kinds of high-frequency changes in the image. On the other hand, it can be omitted while recreating the image to maintain training stability.

Thus, a novel algorithm for SAR imagery Deep Neural Network pretraining was proposed. The input to the network is a simple amplitude SAR image, while the expected output is the same image or one that is processed using simple filtering. The filtering is not about creating a denoising AI model but limiting the influence of random noise/speckle from the original imagery on the AI Loss Function. The filtering algorithm is described in Section 2.6.

### 2.6. Data Preprocessing

The filter types and their parameters depend on the original image parameters. With a higher Signal to Noise Ratio (SNR), the filtering should be weaker.

For the used database, the proposed filtering was in the following order: Gaussian filtering and median filtering with 7 × 7 pixels kernels. Such an approach limits single high-value outliers thanks to Gaussian blurring, and when the median filter is applied the speckle noise is reduced.

It is worth mentioning that the median filter is computationally expensive. Thus, it can be omitted for faster operations or replaced with bilateral filters as proposed in [17].

**Figure 5:**
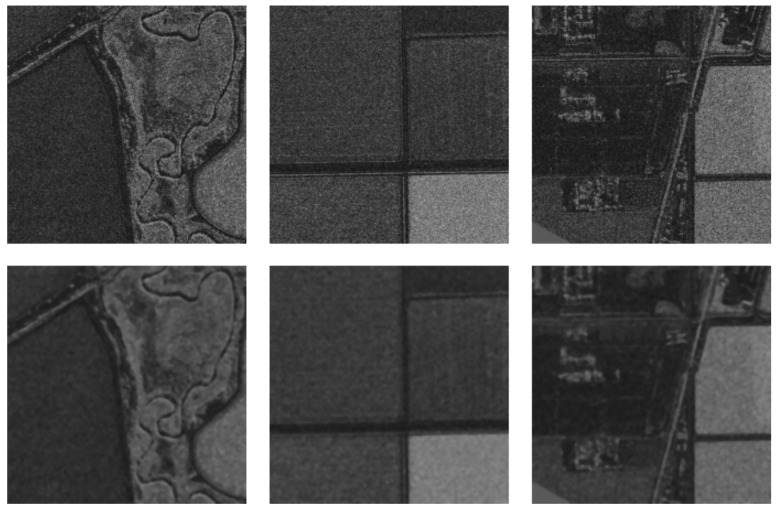
Two pairs of the images before (X—input) and after filtering (Y—labels).

The filtering is shown in Figure 5. The goal was to supply the image without filtering to the neural network to learn, while the filtered image was used as the label (expected output). This way, neural networks should be able to cope with noise/speckle features while being more resistant to noises in the loss determination phase.

## 3. Dataset

The dataset consisted of over 600 high-resolution satellite scans in the X-Band. The dataset was collected from TerraSAR-X working in ScanSAR, Spotlight, and Stripmap modes, made available by the European Space Agency. The orbit height was 514 km at the equator, at an inclination of 97.44°. The Open Data program imagery from Capella Space was also used. The resolution range was between 1 m × 1 m and 20 m × 20 m, and image sizes covered over 10,000 × 20,000 pixels. The scans were gathered from nadir or close to nadir angles.

For training purposes, the images were compressed into 8-bit png images. For Neural Network training, sub-images were extracted with a size of 1024×1024 pixels with the scale N:1 where N ∈ <1, 10>. This was performed as an extraction of a sub-image of size 1024k×1024k pix and zooming out according to the description in the Section 2.1 (where *k* is scaling coefficient, k∈N). Images with a black area of bigger than 20% of the image were deleted from the dataset.

A train-val-test split was performed in the ratio (75%–20%–5%). It is important to mention that the test dataset consisted of all sub-images with 5% of the high-resolution images, so that they did not occur in the training or validation data, while the train-val split was performed in a per-sub-image manner.

## 4. Loss Value Calculation

For image generative neural networks, two main types of Per Pixel Loss Function values were used, namely the Mean Absolute Error (MAE) and Mean Square Error (MSE). They are defined in Equations (1) and (2).

(1) $$\mathrm{MAE}=\frac{\sum_{j=1}^{n}\sum_{i=1}^{n}\left|x\left(i,j\right)-y\left(i,j\right)\right|}{n^{2}}$$

(2) $$\mathrm{MSE}=\frac{\sum_{j=1}^{n}\sum_{i=1}^{n}{\left(x\left(i,j\right)-y\left(i,j\right)\right)}^{2}}{n^{2}}$$

where: *n*—image height and width, x(i,j)y(i,j)—pixels in positon (i,j) of generated and target image.

MSE is more popular, but it focuses on outliers (single high error pixels), which is undesirable in the case of heavily speckled SAR imagery. Additionally, MSE works the best for images with normally distributed values, which requires further image processing. As a result, the MAE loss function was chosen for DNN training.

A different class algorithm of loss value calculation is SSIM. It is a neural network-based algorithm for common feature detection (image similarity). Due to the unusual image type, it does not apply well to SAR imagery. It can be seen in Table 1 and Table 2.

## 5. Proposed Pipeline

**Figure 6:**
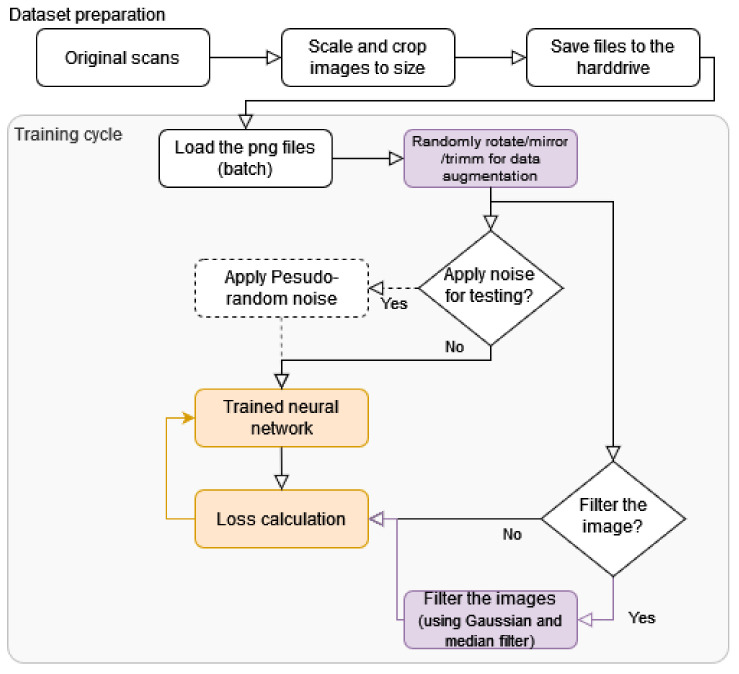
Graph presenting the used pipeline for autoencoders training and testing. Orange color represents training with the backpropagation, purple presents the described processes.

The graph in Figure 6 presents the whole training pipeline. Original scans (in .nitf, .tif and .cos file format) were trimmed to 1024×1024 pixels (including down-scaled from bigger areas) (described in the Section 3). Images prepared in this way are split in train/val/test and saved for all trainings, according to the description in Section 3).

The training process starts with the loading of a batch of images, which are randomly rotated/mirrored and can be trimmed to augment the database (Section 2.1). These images are supplied to the input and output batch generators.

The images are supplied as input (X) for neural network training and images are passed through without modification or a pseudo-random Gaussian noise is applied to them. The purpose of the noise application is to perform statistical analysis (see Section 6.2). The networks were not trained with the noise, as only inference was performed for the algorithm analysis.

The labels or reference values (y) also have the following two paths: pass unmodified to perform classic training, perform filtering for the described training improvement. Passing without any alternation was used to create reference networks, which are juxtaposed in the Results Section. The applied filtering is described in Section 2.6. The filtering improves the achieved metrics as described in the Results Section (Table 1 and Table 2).

## 6. Results

### 6.1. Neural Networks

The codes for models generation in Keras are attached in Appendix B. For feature extraction, a set of neural networks is proposed:

1. Simple CNN,
2. Compressing CNN,

The goal of such a setup is to provide tools for different applications. All the networks were topped with a set of Transposed 2D Convolutional layers as a head. The encoding part of the networks fulfilled the task of the pretext task output. Thus, this part of the neural networks was not researched in the following models. Often, this part of the network is oversized to ensure that it will not affect the efficiency of the network.

Listing defining the architecture of the neural networks is presented in Appendix B.

(A) “Simple CNN” is a set of (3) convolutions with max pooling and dropout. Its goal is to create a possibly wide variety of features for SAR imagery. This allows for the best statistical analysis of the image due to the big feature set, which is calculated quickly.

(B) Compressing CNN is a deeper (5) convolution neural network.This leads to higher compression of the data and more advanced features. It can be used in further processing with more advanced processing due to the lower number of “words” generated.

### 6.2. Comparison of the Model Fitting

Due to the vast dataset, the models were typically trained for two epochs (split into multiple sub-epochs for better performance monitoring). While less than an epoch would often suffice, no overtraining occurred due to almost no image repetition. As a comparison of these model, Table 1 and Table 2 with performance metrics are juxtaposed. Both tables use the same coloring, whereby the green cell represents better metrics (with/without filter pair), and gray numbers underline that the neural network was NOT trained for this operation.

The values in Table 2 present a simple difference between average metric output and the same metric for images with Gaussian noise. The lower the values, the better the result. The output values can be negative, representing occurrence, where noise improves the metrics (this can occur for poorly trained networks).

For time measurement, a computer with Intel Xeon W-2133 CPU was used (3.6 GHz, 12 Cores).

Table 1 and Table 2 indicate the high metrics advantage of the Neural Networks trained with filtered labels over simple autoencoders. The advantage is very interesting considering that in all tests, the metrics values were lower (better) for filter DNN, even where unfiltered images were compared. This might be the case due to a higher needed for “understanding” of the image (the greater neighboring area has to be considered for pixel value decoding) and better normalization.

Another interesting note is that the noise actually has a greater influence on filtered images (Table 2), which lowers the convergence of the learning. This should not result in divergent learning but expends the time needed for learning.

In both tables, an additional CNN with SSIM metric was attached for comparison purposes. As it can be seen, SSIM performed poorly with the SAR imagery.

**Figure 7:**
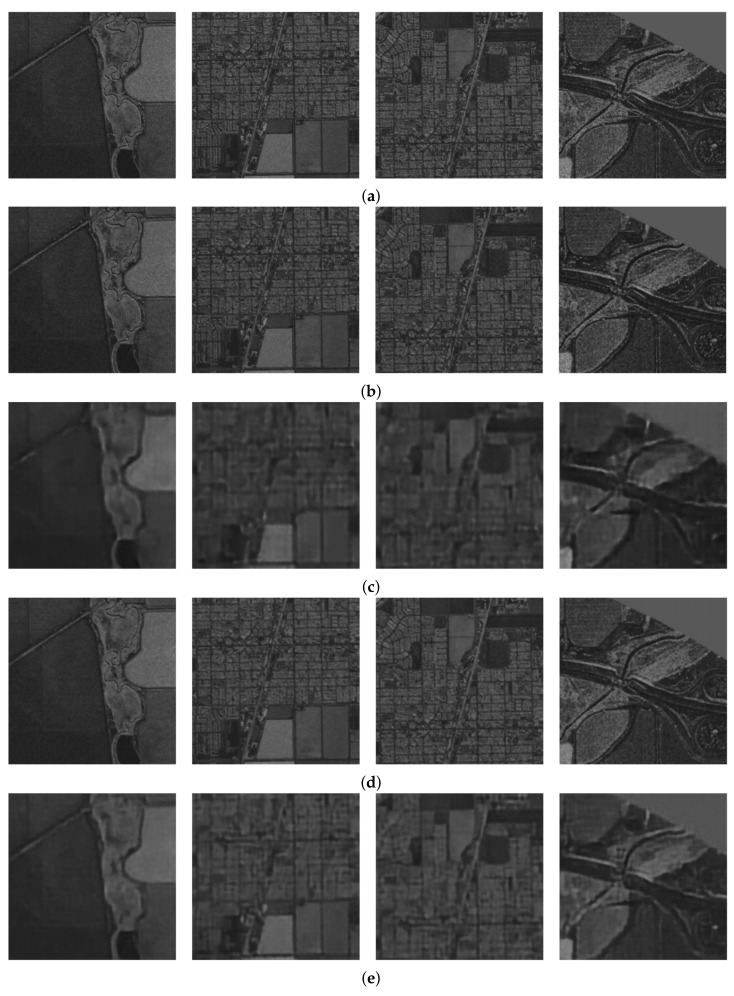
Examples of outputs generated by four neural networks configuration juxtaposed with input images. Outputs of the neural networks learned using filtered labels are slightly less sharp. All the presented networks can compress the data in the images and decode it very well. (**a**) Original Inputs. (**b**) Fully CNN—Without filters. (**c**) Compressing CNN—Without filters. (**d**) Fully CNN—With filters. (**e**) Compressing CNN—With filters.

Example result images for two networks learning with different labels are shown in Figure 7. The first row presents original data put as input to the neural networks. The “Fully CNN—Without filters” replicates the input very accurately. Some minor artefacts can be seen, but it is hard to distinguish this from the original imagery. The “Compressing CNN—Without filters” causes images to blur significantly. The images maintain rough features but loose details.

The last two rows present results for networks trained with filtered labels/reference images. The “Fully CNN” network in this setup does not carry forward noises for the size of individual pixels, but by doing so, it also loses some of the minor features. It is not hard to differentiate it from the original image, but the image itself is still useful for any kind of processing. “Compressing CNN” trained with filters blurs the image more than “Fully CNN.” The image maintains visibly more details than the same network without filtering (row b). This suggests that the neural network with labelled outputs learns better feature sets and thus can replicate the original image more accurately, even though it was trained to match slightly blurred images.

### 6.3. Features and Weights of the Kernels

**Figure A1:**
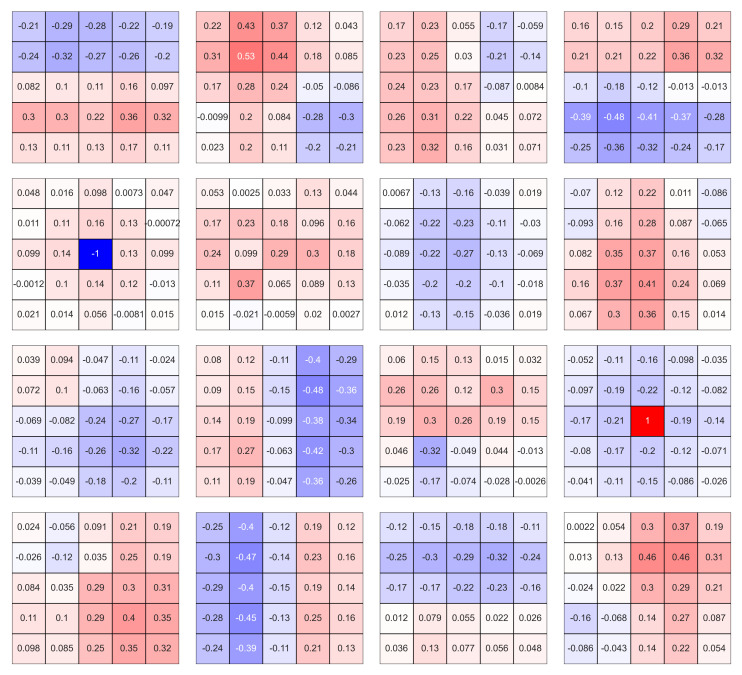
Normalized kernels of 1st layer of Fully CNN with filtered labels.

The neural networks were supplied with displays of the kernel weights of the convolutional neural networks and features displayed for each layer. An example is shown in Figure A1. The kernels were extracted from the trained simple convolutional network’s first layer (index 0) trained with filtering. These are not all the kernels in these layers, but they do represent a very distinct few tendencies of the CNN. The lower layers of the network create features that are angle-invariant. For example, kernels in the first column, second row, and fourth column 3rd row represent quasi-symmetrical functions similar to the sinc function. Other variants of the same/very similar patterns occur in four directions (edge and gradient detectors can be seen easily in this figure).

**Figure A2:**
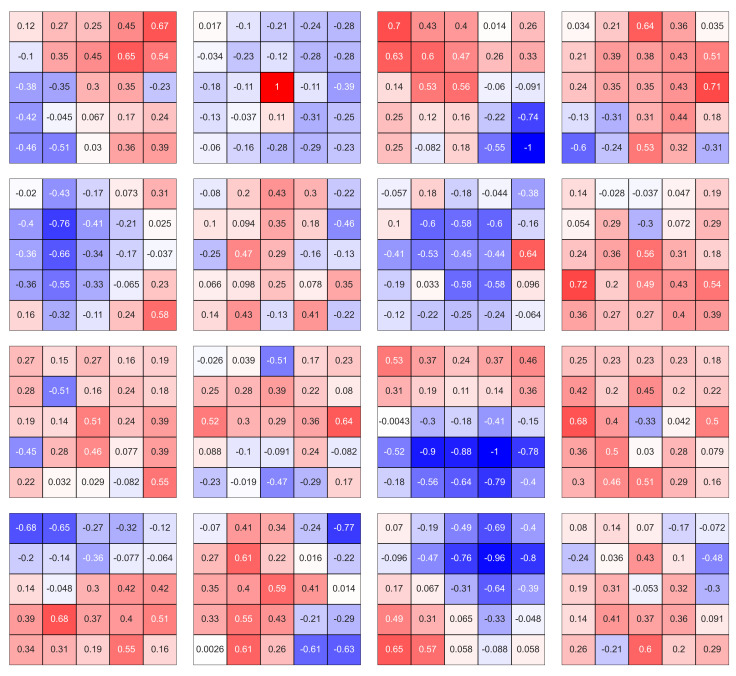
Normalized kernels of 1st layer of Fully CNN with unfiltered labels.

For comparison, a set of weights of a neural network trained without filtered labels is shown in Figure A2. The comparison of the networks is difficult kernel-wise, but an analysis of the kernel weights during different experiments leads to the conclusion that networks with filtered labels create more chaotic weights for a human observer. This can be seen in the attached starting convolutional layers kernels.

**Figure A3:**
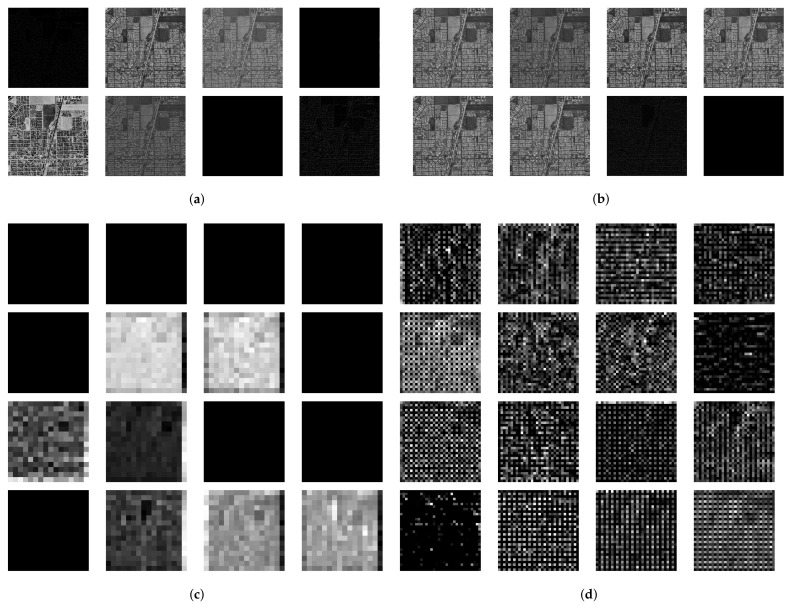
Features extracted from the layers of the Compressing CNN: (**a**) Compressing CNN—Without filters—Layer 0, (**b**) Compressing CNN—With filters—Layer 0, (**c**) Compressing CNN—Without filters—Last coding layer, (**d**) Compressing CNN—With filters—Last coding layer.

Due to the problem with kernel analysis, a set of features for a given image was calculated for each network. For comparison, in Figure A3 the features of the starting and middle layers of ”Compressing CNN” are shown as examples. This setup clearly shows the advantage of the filtered labels as more features are extracted in the deeper layers. This leads to the creation of a better set of features and better image replication.

It is worth noting that in the appliance of the network as a data compressor, the first network would be better suited as a high percentage of the kernels could be pruned, especially if a metric such as L1 or L2 was used during training.

## 7. Comparison to State-of-the-Art Algorithms

Most of the state-of-the-art articles focus on simulated images [33], a deep analysis of complex data [34], or approaches using two or more images of the same area [28]. These approaches require excellent knowledge of the sensor parameters and the platform, including its kinematics/dynamics.

This assumption cannot be taken for most commercial applications, such as Search and Rescue or surveillance missions. This is because airplanes’ or UAVs’ dynamics and paths are heavily weather-dependent. Thus, it is difficult to gather datasets for interferometry-based algorithms [35]. At the same time, simulation-based training requires precise data on the radar build, which is part of the radar system suppliers’ intellectual property and is hardly ever shared.

For these reasons, the paper focused purely on training with real data. A single UAV mission (4–8 h) can generate hundreds of scans. The same amount of data takes days of work for the professional analyst to label. Thanks to a more universal approach focused on analyzing a single final scan, virtually any scans gathered by the system can be used for pre-training. There is no need to prepare special trajectories or missions in favorable atmospheric conditions for dataset gathering.

The goal of the article was to present a lightweight algorithm (both in terms of processor load and implementation requirements) for boosting the effectiveness of autoencoder pretraining. As presented in Section 6, autoencoding capabilities noticeably improved thanks to label image filtering. As such, any quantitative comparison to the cited articles is inappropriate. This is the reason why the performances of the networks with the modified training algorithm are juxtaposed with classic training for comparison purposes. The networks are not supposed to be better at autoencoding/filtering than those of the cited articles but provide a Bag of Words for transfer learning or statistical analysis (e.g., clustering [36]).

## 8. Conclusions

This article presents and justifies different tools for data augmentation and autoencoder (or GAN) neural networks training. Additionally, the training source codes and the trained neural networks are attached to a proper repositories.

The novel method of image filtering for the autoencoders provides better neural network pretraining. Thanks to simple blurring algorithms (Gaussian blur and median filtering), the operations are fast and easy to perform, while the gain in the metrics varies from 20 to 50%! The algorithm is also very easy to implement in a real deployment environment, as it does not use complex data or correlated imagery. The only cost of the algorithm is a higher CPU load during the image pre-processing.

Neural networks created in this way are perfect for transfer learning or further statistical or analytical processing. The networks presented here are convolutional, which allows for very quick calculations using GPU, but times under 250 ms are achieved with PC class CPU. Additionally, proper pruning of the given neural networks can be performed depending on the requirements. It was not performed to allow for the fine-tuning of the neural networks for the final task or the use of more advanced methods of pruning [37].

In the future, other architectures and neural network types (such as GANs) could be tested by implementing the proposed image pre-processing.

## Figures and Tables

**Table 1:** Comparison of Neural network performance over test set. Green background determines the better results, while gray font means that the neural network was not trained for this task (filtered versus unfiltered output).

Neural Network		Inference Time [s]	Unfiltered		Filtered		Noised Unfiltered		Noised Filtered	
			MSE	MAE	MSE	MAE	MSE	MAE	MSE	MAE
Compressing CNN	Without filters	0.237	0.0382	0.1493	0.0030	0.0410	0.0408	0.1552	0.0407	0.1549
	With 7 × 7 Filter	0.234	0.0366	0.1478	0.0018	0.0305	0.0368	0.1482	0.0367	0.1480
Fully CNN	Without filters	0.246	0.0333	0.1380	0.0025	0.0372	0.0352	0.1424	0.0353	0.1424
	With 7 × 7 Filter	0.249	0.0329	0.1372	0.0003	0.0128	0.0344	0.1421	0.0344	0.1430
Compressing CNN with SSIM metric	Without filters	0.238	0.1924	0.3557	0.1575	0.3548	0.1925	0.3559	0.1925	0.3559
	With × 7 Filter	0.235	0.0653	0.1908	0.0306	0.1441	0.0563	0.1792	0.0569	0.1802

**Table 2:** Simple analysis of the error change due to noise application. Green background determines the better results, while gray font means that the neural network was not trained for this task.

Neural Network		Effect of Noise on			
		Unfiltered MSE	Unfiltered MAE	Filtered MSE	Filtered MAE
Compressing CNN	Without filters	0.0026	0.0059	0.0376	0.1139
	With × 7 Filter	0.0002	0.0004	0.0349	0.1175
Fully CNN	Without filters	0.0020	0.0044	0.0327	0.1053
	With × 7 Filter	0.0005	0.0014	0.0341	0.1302
Compressing CNN with SSIM metric	Without filters	0.0001	0.0001	0.0349	0.0011
	With × 7 Filter	−0.0090	−0.0116	0.0263	0.0361

## Data Availability

The authors would like to thank European Space Agency for access to the TerraSAR-X database and Capella Space Program for Open Data Imagery used as examples in the article.

## Source Codes

Source codes available on Github service under link: https://github.com/Krisequ/-sar_filtered_labels_experiment.

## AI Models

The model weights are available for download under the link attached in the README.md of the GitHub repository above.

## Abbreviations

The following abbreviations are used in this manuscript:

AI	Artificial Intelligence
CNN	Convolutional Neural Networks
GAN	Generative Adversarial Network
DNN	Deep Neural Networks
MAE	Mean Absolute Error
MSE	Mean Square Error
SAR	Synthetic Aperture Radar
SNR	Signal to Noise Ratio
SSIM	Structural Similarity
YOLO	You Only Look Once—Neural Network Family

## Appendix B. Definition of the Used Convolution Neural Networks

**Listing A1.** Factory functions definitions-Python 3, Keras 2.7.0.

## References

[1] Le N.Q.K., Ho Q.T. (2022). Deep transformers and convolutional neural network in identifying DNA N6-methyladenine sites in cross-species genomes. Methods (San Diego Calif.).

[2] ImageNet. https://www.image-net.org/.

[3] COCO—Common Objects in Context. https://cocodataset.org/#home.

[4] CIFAR-10 and CIFAR-100 Datasets. http://www.cs.toronto.edu/~kriz/cifar.html.

[5] GitHub—v7labs/COVID-19-Xray-Dataset: 12000+ Manually Drawn Pixel-Level Lung Segmentations, with and without COVID. https://github.com/v7labs/covid-19-xray-dataset.

[6] FREE—FLIR Thermal Dataset for Algorithm Training|Teledyne FLIR. https://www.flir.com/oem/adas/adas-dataset-form/.

[7] Jiang P., Ergu D., Liu F., Cai Y., Ma B. (2022). A Review of Yolo Algorithm Developments. Procedia Comput. Sci..

[8] Allen-Zhu Z., Li Y. (2019). What Can ResNet Learn Efficiently, Going Beyond Kernels?. Adv. Neural Inf. Process. Syst..

[9] DeepLearningExamples/PyTorch/Classification/ConvNets/resnet50v1.5 at Master · NVIDIA/DeepLearningExamples. https://github.com/NVIDIA/DeepLearningExamples/tree/master/PyTorch/Classification/ConvNets/resnet50v1.5.

[10] He K., Gkioxari G., Dollár P., Girshick R. (2020). Mask R-CNN. IEEE Trans. Pattern Anal. Mach. Intell..

[11] Natteshan N., Kumar N.S. (2020). Effective SAR image segmentation and classification of crop areas using MRG and CDNN techniques. Eur. J. Remote Sens..

[12] Bovenga F., Pasquariello G., Refice A. (2021). Statistically-Based Trend Analysis of MTInSAR Displacement Time Series. Remote Sens..

[13] Chen S., Wang H., Xu F., Jin Y.Q. (2016). Target Classification Using the Deep Convolutional Networks for SAR Images. IEEE Trans. Geosci. Remote Sens..

[14] Stecz W., Gromada K. (2020). UAV Mission Planning with SAR Application. Sensors.

[15] Richter N., Froger J.L. (2020). The role of Interferometric Synthetic Aperture Radar in Detecting, Mapping, Monitoring, and Modelling the Volcanic Activity of Piton de la Fournaise, La Réunion: A Review. Remote Sens..

[16] Preiss M., Stacy N.J.S. (2006). Coherent Change Detection: Theoretical Description and Experimental Results.

[17] Siemia̧tkowska B., Gromada K. (2021). A New Approach to the Histogram-Based Segmentation of Synthetic Aperture Radar Images. J. Autom. Mob. Robot. Intell. Syst..

[18] Chechliński L., Siemia̧tkowska B., Majewski M. (2019). A System for Weeds and Crops Identification—Reaching over 10 FPS on Raspberry Pi with the Usage of MobileNets, DenseNet and Custom Modifications. Sensors.

[19] Shakya A., Biswas M., Pal M. (2021). Fusion and classification of multi-temporal SAR and optical imagery using convolutional neural network. Int. J. Image Data Fusion.

[20] Shermeyer J., Hogan D., Brown J., Etten A., Weir N., Pacifici F., Hänsch R., Bastidas A., Soenen S., Bacastow T. SpaceNet 6: Multi-Sensor All Weather Mapping Dataset. Proceedings of the 2020 IEEE/CVF Conference on Computer Vision and Pattern Recognition Workshops (CVPRW).

[21] Zhang T., Zhang X., Li J., Xu X., Wang B., Zhan X., Xu Y., Ke X., Zeng T., Su H. (2021). SAR Ship Detection Dataset (SSDD): Official Release and Comprehensive Data Analysis. Remote Sens..

[22] MSTAR Overview. https://www.sdms.afrl.af.mil/index.php?collection=mstar.

[23] Moreira A., Prats-Iraola P., Younis M., Krieger G., Hajnsek I., Papathanassiou K.P. (2013). A tutorial on synthetic aperture radar. IEEE Geosci. Remote Sens. Mag..

[24] Thrower N.J.W., Jensen J.R. (1976). The orthophoto and orthophotomap: Characteristics, development and application. Am. Cartogr..

[25] ICEYE Unveils 25 cm SAR Imaging Capability with Current SAR Satellite Constellation. https://www.iceye.com/press/press-releases/iceye-unveils-25-cm-sar-imaging-capability-with-current-sar-satellite-constellation.

[26] Gromada K., Siemia̧tkowska B., Stecz W., Płochocki K., Woźniak K. (2022). Real-Time Object Detection and Classification by UAV Equipped With SAR. Sensors.

[27] Tran P.V. (2019). Exploring Self-Supervised Regularization for Supervised and Semi-Supervised Learning. arXiv.

[28] Ben Abbes A., Jarray N. (2022). Unsupervised self-training method based on deep learning for soil moisture estimation using synergy of sentinel-1 and sentinel-2 images. Int. J. Image Data Fusion.

[29] He K., Chen X., Xie S., Li Y., Dollár P., Girshick R. (2021). Masked Autoencoders Are Scalable Vision Learners. arXiv.

[30] Ng A. (2011). Sparse autoencoder. CS294A Lect. Notes.

[31] Creswell A., White T., Dumoulin V., Arulkumaran K., Sengupta B., Bharath A.A. (2018). Generative adversarial networks: An overview. IEEE Signal Process. Mag..

[32] Zhang Q., Sun R. (2020). SAR Image Despeckling Based on Convolutional Denoising Autoencoder. arXiv.

[33] Bhamidipati S.R.M., Srivatsa C., Kanakapura Shivabasave Gowda C., Vadada S. (2020). Generation of SAR Images Using Deep Learning. SN Comput. Sci..

[34] Hirose A., Sunaga Y., Natsuaki R. Recent progress in adaptive sar data structurization in complex and hypercomplex domain. Proceedings of the 2019 SAR in Big Data Era, BIGSARDATA 2019.

[35] Stecz W., Gromada K. (2020). Determining UAV flight trajectory for target recognition using EO/IR and SAR. Sensors.

[36] Ferreira N., Silveira M. Ship Detection in SAR Images Using Convolutional Variational Autoencoders. Proceedings of the International Geoscience and Remote Sensing Symposium (IGARSS).

[37] Yeom S.K., Seegerer P., Lapuschkin S., Binder A., Wiedemann S., Müller K.R., Samek W. (2021). Pruning by explaining: A novel criterion for deep neural network pruning. Pattern Recognit..

